# Target RNAs Strike Back on MicroRNAs

**DOI:** 10.3389/fgene.2018.00435

**Published:** 2018-10-02

**Authors:** Federico Fuchs Wightman, Luciana E. Giono, Juan Pablo Fededa, Manuel de la Mata

**Affiliations:** ^1^Facultad de Ciencias Exactas y Naturales, Universidad de Buenos Aires, Buenos Aires, Argentina; ^2^Consejo Nacional de Investigaciones Científicas y Técnicas-Universidad de Buenos Aires, Instituto de Fisiología, Biología Molecular y Neurociencias, Buenos Aires, Argentina; ^3^Instituto de Investigaciones Biotecnológicas, Universidad Nacional de San Martín, Consejo Nacional de Investigaciones Científicas y Técnicas, Buenos Aires, Argentina

**Keywords:** microRNA, degradation, tailing and trimming, uridylation, TDMD, Argonaute, exoribonuclease, terminal nucleotidyl transferase

## Abstract

MicroRNAs are extensively studied regulatory non-coding small RNAs that silence animal genes throughout most biological processes, typically doing so by binding to partially complementary sequences within target RNAs. A plethora of studies has described detailed mechanisms for microRNA biogenesis and function, as well as their temporal and spatial regulation during development. By inducing translational repression and/or degradation of their target RNAs, microRNAs can contribute to achieve highly specific cell- or tissue-specific gene expression, while their aberrant expression can lead to disease. Yet an unresolved aspect of microRNA biology is how such small RNA molecules are themselves cleared from the cell, especially under circumstances where fast microRNA turnover or specific degradation of individual microRNAs is required. In recent years, it was unexpectedly found that binding of specific target RNAs to microRNAs with extensive complementarity can reverse the outcome, triggering degradation of the bound microRNAs. This emerging pathway, named TDMD for Target RNA-Directed MicroRNA Degradation, leads to microRNA 3′-end tailing by the addition of A/U non-templated nucleotides, trimming or shortening from the 3′ end, and highly specific microRNA loss, providing a new layer of microRNA regulation. Originally described in flies and known to be triggered by viral RNAs, novel endogenous instances of TDMD have been uncovered and are now starting to be understood. Here, we review our current knowledge of this pathway and its potential role in the control and diversification of microRNA expression patterns.

## Introduction

Since their discovery 25 years ago, metazoan microRNAs (miRNAs) have been undisputedly recognized as fundamental actors in regulation of gene expression, providing a layer of control at the posttranscriptional level which complements and builds upon other layers of gene regulation. Recently described as “Sculptors of the Transcriptome,” miRNAs typically act by exerting a subtle silencing or fine tuning of mRNAs delivered to the cytoplasm by the transcriptional and RNA processing nuclear machineries (Bartel, 2018). In the last two decades much has been learned about miRNA mechanisms of action, evolution, and their pathophysiological roles. Indeed, from their emergence in the first multicellular organisms (Grimson et al., 2008), miRNAs have played key roles in their development and homeostasis (Bartel, 2018). Because miRNAs themselves are differentially expressed between different cell types and across development, a large proportion of studies about miRNAs has focused on their regulation at the level of transcription and biogenesis (**Box 1**). However, relatively less attention has been given to the mechanisms of miRNA turnover. This broadly obeys to the fact that miRNAs tend to be generally stable in various cell types, with half-lives extending to days (Hwang et al., 2007; van Rooij et al., 2007; Gatfield et al., 2009; Bail et al., 2010; Baccarini et al., 2011; Gantier et al., 2011; Guo et al., 2015). In fact, in many cases where miRNAs and mRNAs are produced from common primary RNAs, it is not rare to observe much higher levels of the miRNA than of the cotranscribed mRNA. In this sense, even when part of the observed differences in abundance could arise from differences in the efficacy or competition between the biogenesis steps that produce the different RNA species, increased stability of miRNAs seems to be the prevalent cause (Bell et al., 2010; Agranat-Tamir et al., 2014; Bartel, 2018).

**Box 1. miRNA source, biogenesis and mode of action.**

The main source of mature miRNAs are the introns or exons of non-coding primary transcripts synthesized by RNA polymerase II (Cai et al., 2004; Lee et al., 2004). However, some miRNA families, like *Mir449a-c*, are derived from introns of coding transcripts (Lagos-Quintana et al., 2001; Lau et al., 2001). The usually long primary-microRNAs (pri-miRNAs) generate a secondary hairpin structure, which is recognized by the microprocessor complex in the nucleus, formed by Drosha and DGCR8. The RNASE III catalytic activity of Drosha processes pri-miRNA hairpins into smaller precursor stem-loops called pre-miRNAs, leaving 1-nt or 2-nt 3′ overhang structures (group I or II miRNAs, respectively) (Lee et al., 2003). Pre-miRNAs are then exported to the cytoplasm by Exportin-5 (Yi et al., 2003; Bohnsack et al., 2004; Lund et al., 2004), and further processed by Dicer in the cytoplasm (Bernstein et al., 2001; Grishok et al., 2001; Hutvagner et al., 2001; Ketting et al., 2001). Dicer selectively recognizes pre-miRNAs by their 2-nt 3′ overhang (Zhang et al., 2004) and cuts both strands of the pre-miRNA stem allowing the release of the loop. The remaining miRNA duplex is loaded into the AGO protein with the help of HSC70/HSP90 (Iwasaki et al., 2010; Miyoshi et al., 2010). The passenger strand of the duplex (also called miRNA^∗^) then is expelled and degraded, and AGO adopts a stable RISC (RNA inducible silencing complex) conformation with the remaining mature miRNA guide strand (Khvorova et al., 2003; Liu et al., 2003; Tomari et al., 2004). There are exceptions where both strands of the miRNA duplex are able to load into a mature RISC complex. Non-canonical biogenesis pathways are adopted by a special type of miRNAs encoded in full introns (or “mirtrons”), bypassing the microprocessor complex, or by certain miRNAs like miR-451, which detours Dicer processing (Ha and Kim, 2014). To enforce silencing in bilaterians, mature RISC “scans” mRNA transcripts guided by the sequence of the loaded miRNA. Target sites are recognized primarily by sequence pairing with the “seed” region, comprising positions 2–7 of miRNA’s 5′ end (Bartel, 2009). Once a target site is recognized, most commonly in the 3′-UTR of the target transcript, RISC recruits GW182, which interacts with PABPC (Eulalio et al., 2008; Fabian et al., 2009). These interactions trigger the recruitment of deadenylase complexes CCR4-NOT or PAN2–PAN3, which shorten the poly A tail of the target mRNA (Yamashita et al., 2005; Wahle and Winkler, 2013; Jonas and Izaurralde, 2015). Depending on the physiological context, poly A shortening induces translation inhibition, or decapping and degradation of the target mRNAs (Jonas and Izaurralde, 2015).

Despite miRNAs being truly stable in many systems and conditions, certain developmental transitions and stimuli in some cell types sometimes lead to a sudden drop in the concentration of specific miRNAs. For instance, miRNAs are substantially less stable in neurons (Rajasethupathy et al., 2009; Sethi and Lukiw, 2009; Krol et al., 2010a), during specific stages of the cell cycle (Rissland et al., 2011), and during viral infections (Buck et al., 2010; Cazalla et al., 2010; Rissland et al., 2011; Marcinowski et al., 2012; Lee et al., 2013), all cases where active miRNA degradation has been demonstrated (reviewed in Ruegger and Grosshans, 2012). This is in line with the notion that, as posttranscriptional regulators, miRNAs are well suited for induction of rapid and spatially localized changes in gene expression. Accordingly, dynamic changes in target gene activity in response to variations in miRNA biogenesis rates must require mechanisms of active and regulated miRNA turnover (Hausser et al., 2013). Nevertheless, the general physiological mechanisms of miRNA degradation are not fully understood. In particular, an intriguing aspect of miRNA turnover is how individual miRNAs are selectively discriminated among other miRNAs and especially from sequence-related family members. Together with the limited targeting specificity of RNA binding proteins (RBPs) on small sized miRNA, this makes the selective miRNA degradation a mechanistic challenge. In this scenario, growing evidence suggests that specific RNA targets themselves can direct specific miRNAs for degradation, reverting the direction of the canonical miRNA pathway. In this review, we describe the findings related to TDMD and discuss the potential implications in the homeostasis of miRNAs with a focus on mammalian cells.

## General Features of TDMD

**FIGURE 1:**
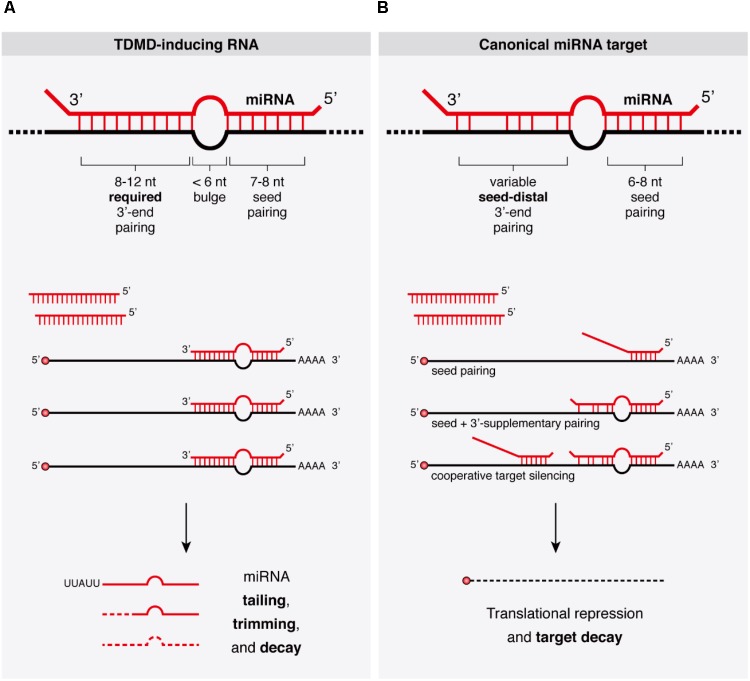
Target binding architectures define the outcomes of miRNA binding. **(A)** Properties of TDMD-inducing target RNAs bound to their cognate miRNAs and concomitant miRNA tailing and trimming species accumulating during TDMD are schematically represented. Substoichiometric target RNA concentrations may suffice to trigger TDMD, allowing miRNAs to induce only limited or no target degradation. **(B)** Properties of canonical miRNA targets typically leading to target RNA silencing upon miRNA binding. Additional architectures such as ‘centered’ or ‘seedless’ interactions have been omitted, though the latter has been linked to TDMD (see text and **Figure 2B**).

The discovery that target RNAs themselves can trigger specific miRNA degradation in animal cells through the process of TDMD was first described mechanistically in *Drosophila* and in mammalian cells (Ameres et al., 2010). During TDMD, the downregulation of miRNAs involves neither a decrease in transcription of miRNA genes nor an inhibition of the processing of primary or precursor miRNA (pri- or pre-miRNA, respectively), but rather an active degradation of mature miRNAs after they are loaded into Argonaute (AGO) (Cazalla et al., 2010; de la Mata et al., 2015; Kleaveland et al., 2018). As opposed to the canonical function of miRNAs in target silencing, TDMD is characterized by a different miRNA–target architecture that enables target RNAs to evade silencing in favor of a destabilization of the bound miRNAs, and seems to display differences in efficacy among different cell types. The extent of sequence complementarity between miRNA and mRNA and their relative abundances define the possible outcomes: whereas only partial pairing minimally encompassing the 5′ seed region of miRNAs is sufficient to exert silencing of the targeted mRNAs through translational repression and mRNA decay (**Box 1**) (Bartel, 2018), a more extensive pairing through the 3′ region of the miRNA seems to be required for TDMD (**Figure 1**). Interestingly, TDMD is conserved in animals, and although natural TDMD-inducing targets seem to be rare or difficult to predict, endogenous instances of TDMD are beginning to emerge.

## Inducers of TDMD

### Artificial Target RNAs

The discovery that specific miRNA–target binding architectures in the context of capped and polyadenylated target mRNAs can trigger miRNA degradation in animal cells came from the Zamore lab who brought the first mechanistic insight into TDMD (Ameres et al., 2010). This finding originated from the observation that, unlike siRNAs, *Drosophila* miRNAs rarely show extensive base pairing to the mRNAs they regulate, but when exposed to extensively complementary artificial target RNAs, miRNAs are efficiently degraded. More generally, it was shown that, both in *Drosophila* and in mammalian cells, binding of miRNAs to RNAs with extensive complementarity triggered miRNA 3′-end tailing (addition of non-templated nucleotides, usually U’s or A’s), trimming (shortening from the 3′ end), and degradation of the mature miRNA (**Figure 1**). Subsequent kinetic studies extended these results by showing that extensively paired artificial target RNAs accelerated the actual rate of miRNA decay in mammalian cells, while also increasing the frequency of posttranscriptional addition of non-templated uridines to miRNAs (Baccarini et al., 2011).

**FIGURE 2:**
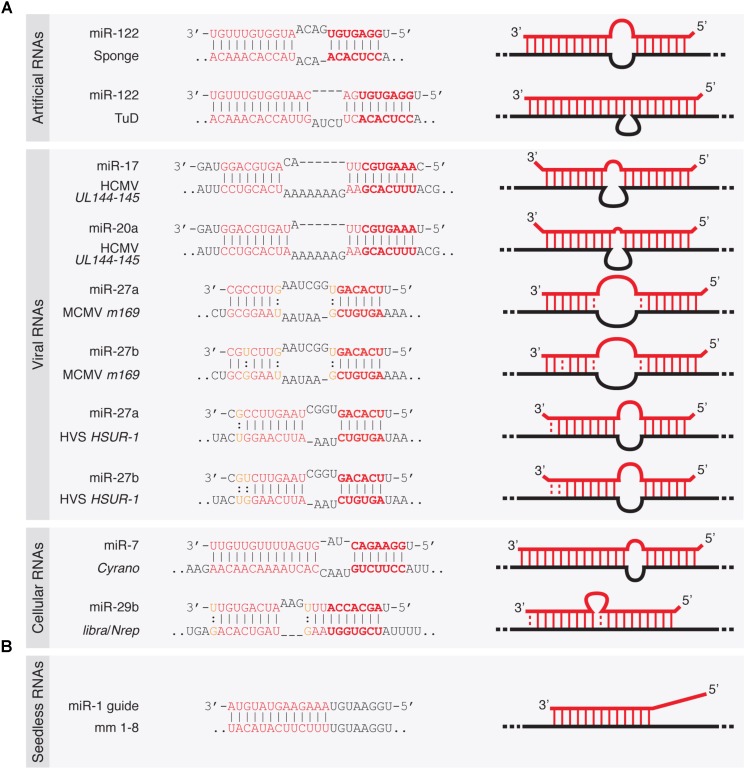
Architecture of TDMD-inducing miRNA binding sites described in the literature. **(A)** Seed containing miRNA binding sites within artificially designed or naturally occurring target RNAs—either of viral or cellular origin. **(B)** Seedless miRNA binding site present in an artificial target RNA shown to elicit TDMD *in vitro* (Park et al., 2017). Dashes represent Watson–Crick base pairs and dots represent Wobble base pairs.

Artificial target mRNAs with TDMD-competent binding site architectures had been tested previously within the so-called miRNA “sponges” (Care et al., 2007; Ebert et al., 2007; Gentner et al., 2009) (**Figure 2A**). RNA polymerase II-transcribed miRNA sponges were originally conceived as genetic tools to block miRNA activity *in vivo* by acting as competitive inhibitors of miRNAs inside the cell, where they could be stably integrated into the genome, thus allowing the creation of stable cell lines and transgenic animals functionally deficient for a specific miRNA family. However, while miRNA sponges effectively blocked miRNA activity—presumably by sequestering miRNAs—, they proved to have relatively little effect on miRNA stability (Ebert et al., 2007). It was later shown that artificial sponge-like target architectures generally induce a relatively modest TDMD effect in mammalian non-neuronal cell lines, while they exhibit a much more potent TDMD in neuronal cells (de la Mata et al., 2015; Kleaveland et al., 2018). Other artificial targets such as tough decoys (TuDs) do achieve high degradation efficacies of up to fivefold in non-neuronal cells by means of an optimized miRNA–target architecture and the strong RNA polymerase III U6 promoter (Xie et al., 2012). This is consistent with the first reports describing an efficient targeted miRNA degradation upon binding to antisense “antagomirs,” namely short, chemically modified, antisense oligonucleotides typically used as miRNA inhibitors at high numbers *in vivo* (Krutzfeldt et al., 2005, 2007). The apparent variability of TDMD efficacy induced by different artificial targets might be therefore explained by a number of reasons which include cell-type specificity, miRNA–target architecture and relative miRNA–target abundance (discussed below), although a clearer picture of the minimum requirements for efficacious TDMD remains to be determined.

### Viral Target RNAs

Viruses employ different strategies to affect gene expression of the host, and typically co-opt cellular machinery for their own benefit. Some viral families achieve that through virally encoded miRNAs while others also block host miRNAs through anti-miRNA activities (Guo and Steitz, 2014). Accordingly, it has been shown that certain viruses take advantage of TDMD through viral RNAs that can destabilize certain miRNAs of the host (Guo and Steitz, 2014; McCaskill et al., 2015). This was first described for the *Herpesvirus saimiri* (HVS), an oncogenic gamma-herpesvirus targeting miR-27 for degradation (Cazalla et al., 2010). HVS expresses seven U-rich small non-coding RNAs (ncRNAs) called HSURs, structurally related to small nuclear RNAs (snRNAs). In virally transformed primate T cells, *HSUR-1* and -*2* bind the partially complementary host miRNAs miR-142-3p, miR-27 (comprising miR-27a and miR-27b, which differ by one nucleotide), and miR-16. Of these, miR-27 exhibits the most extensive base pairing with *HSUR-1* and is destabilized by this interaction (**Figure 2A**). This in turn increases *FOXO1* levels, a validated miR-27 target. By contrast, the levels of miR-142-3p or miR-16 are refractory to degradation by HSURs, consistent with a less extensive base-pairing.

Like HVS, murine cytomegalovirus (MCMV, a beta-herpesvirus) triggers a rapid downregulation of the host’s miR-27 through the viral transcript *m169*, by pairing to an extensively complementary miR-27 binding site in its 3′-untranslated region (UTR) (Libri et al., 2012; Marcinowski et al., 2012) (**Figure 2A**). The MCMV transcriptome bears hundreds of potential miR-27-binding sites, but only m169 is responsible for miR-27 degradation (Libri et al., 2012). More interestingly, expression of *m169* 3′-UTR alone in uninfected cells is sufficient for miR-27 degradation, revealing that the whole TDMD degradation machinery is provided by the host, with no additional viral factors required other than a single RNA target as the trigger (Marcinowski et al., 2012). Consistent with previous reports, miR-27 degradation occurred concomitantly with miRNA tailing and trimming, and both processes were dependent on an intact miR-27 binding site in *m169* (Marcinowski et al., 2012). How do these viruses benefit from miR-27 degradation? Despite regulating the same miRNA, the operating mechanisms between the different herpesviruses subfamilies seem to differ. For gamma-herpesvirus (including HSV), since miR-27 silences genes that mediate T-cell activation—key for virus propagation in these cells—, acquiring TDMD on miR-27 might have been beneficial for virus spreading. This is supported by the fact that two other gamma-herpesvirus family members (Alcelaphine herpesvirus 1 and Ovine herpesvirus 2) lack a target RNA that downregulates miR-27 but instead encode homologs of miR-27 target genes, thereby bypassing the need to reduce miR-27 levels in the host (Guo et al., 2014). The benefit of degrading miR-27 is less clear for beta-herpesvirus (including MCMV), but because MCMV has different cell tropism than HSV (it infects macrophages, dendritic cells, fibroblasts, and hepatocytes rather than T-cells) (Hsu et al., 2009), the benefit of degrading miR-27 must involve a different mechanism than activating T-cells. As previously suggested, degrading miR-27 might be important for both beta- and gamma-herpesviruses by preventing the silencing of interleukin 10 (IL-10), an immunosuppressive cytokine that is a target of miR-27 (Guo et al., 2014). The fact that many herpesviruses encode viral homologs of IL-10 with similar functions to cellular IL-10 indeed supports this possibility. In this way, herpesviruses might have independently developed TDMD on specific miRNAs or acquired copies of the host protein-coding genes targeted by those miRNAs, thereby modifying the host cell expression program for their own benefit (Guo and Steitz, 2014; McCaskill et al., 2015).

Infection by the human cytomegalovirus (HCMV) represents another case of TDMD triggered by viruses. Upon transduction, two specific miR-17 family members (miR-17 and miR-20a), which are expressed from the *Mir17-92* cluster (encoding six miRNAs: miR-17, miR-18a, miR-19a, miR-20a, miR-19b, and miR-92a), are post-transcriptionally downregulated by TDMD (Lee et al., 2013). Degradation is induced by miRNA binding to a ncRNA region (termed miRDE, for “miRNA decay element”) within the bicistronic *UL144-145* viral mRNA. Similarly to miR-27 degradation by HVS and MCMV, the miRDE presents specific and extensive base pairing to miR-17 and miR-20a (**Figure 2A**). Although currently unclear, miR-17 and miR-20a degradation appears to benefit HCMV DNA synthesis, leading to a higher viral production during infection. This is evidenced by the fact that HCMV strains carrying mutations in the single miR-17/miR-20a binding site show reduced synthesis of viral DNA and delayed viral production during lytic infection. The functional relevance of TDMD in regulating the miR-17 family is reinforced by the fact that the sequence of the TDMD-inducing element is perfectly conserved among all HCMV clinical isolates. Moreover, the virulence of HCMV correlates with the presence of this element in the viral genome, and its absence in attenuated laboratory strains (Lee et al., 2013). The host *Mir17-92* cluster, also known as OncomiR-1, and its paralogs (*Mir106a-363* and *Mir106b-25* clusters) act as oncogenes (Mogilyansky and Rigoutsos, 2013). However, since HCMV is not oncogenic, how the degradation of these host miRNAs within the *Mir17-92* cluster is beneficial to the virus remains to be determined (Guo and Steitz, 2014).

It is currently unknown how widespread TDMD might be among other virus families. However, when considering that individual cellular miRNAs are known to interfere with specific viruses during viral infection or replication, it is conceivable that other viruses might have taken advantage of TDMD as an anti-miRNA activity (McCaskill et al., 2015). A more comprehensive understanding of the TDMD phenomenon is still needed to reach a wider and deeper knowledge of its implications for viral infections.

### Cellular Target RNAs

Despite the potency of artificial and viral targets in triggering miRNA degradation and the conservation of TDMD activity in different species, its physiological relevance has remained questionable. This is in part due to the apparent lack in metazoan genomes of highly complementary miRNA binding sites that would typically elicit TDMD. However, recent reports have found cellular RNAs that trigger degradation of miRNAs through TDMD which are associated with observable phenotypes. The endogenous transcripts *libra* and its homolog *Nrep* in the cerebellums of zebrafish and mouse, respectively, have been recently shown to trigger decay of miR-29b, one of the three members of the miR-29 family (Bitetti et al., 2018) (**Figure 2A**). In turn, impairment of miR-29b degradation by TDMD causes aberrant explorative and anxiety-like behaviors both in fish and in mice. These transcripts appear to be evolutionarily related, but while the zebrafish *libra* transcript is a long non-coding RNA (lncRNA), mouse *Nrep*, as well as its human homolog, is an mRNA encoding a small protein. A perfectly conserved 20-nucleotide site complementary to miR-29b in the non-coding 3′-UTR of *Nrep* triggers an efficient TDMD on miR-29b, restricting its spatial expression in the cerebellum. Like viral TDMD-inducing transcripts, the *Nrep* site is characterized by extensive complementarity to the 5′- and 3′-ends with a central 3-nucleotide mismatch (**Figure 2A**). Remarkably, scrambling this site recapitulates the *Nrep* knockout (KO) behavioral phenotypes without affecting *Nrep* mRNA and presumably protein levels. These findings provided the first evidence that TDMD accounts for at least some of the behavioral functions of an endogenous vertebrate gene.

More recently, the lncRNA *Cyrano* has been shown to trigger 3′-end tailing, trimming and decay of miR-7 by means of an extensively paired site present within a short region of high sequence conservation among vertebrates (Kleaveland et al., 2018) (**Figure 2A**). *Cyrano*, a previously proposed decoy for miR-7 (Smith et al., 2017), is a key regulator in zebrafish brain development and is enriched in the early central and peripheral nervous systems in mouse and humans (Ulitsky et al., 2011). Despite miR-7’s reported role in neural differentiation (Cui et al., 2014), *Cyrano* KO mice do not present any obvious abnormalities. Yet, they display striking molecular phenotypes (Kleaveland et al., 2018). Either a full deletion (*Cyrano*^-/-^ animals) or smaller deletions encompassing the seed-binding region of *Cyrano* cause a sharp increase of miR-7 levels in several tissues due to abolished TDMD, particularly in the brain. In turn, this correlates with a modest but significant increase in repression of miR-7 predicted targets in some tissues (**Box 2**). Interestingly, among the miR-7 targets, the one most prominently reduced by *Cyrano* depletion was *Cdr1as*, a circRNA bearing multiple (∼130) binding sites for miR-7. *Cdr1as* also contains a conserved single miR-671 site with sufficient complementarity to allow AGO2-catalyzed slicing of the circRNA (Hansen et al., 2011). Further analysis of the molecular alterations induced by *Cyrano* depletion revealed the existence of a regulatory network consisting of four ncRNAs: the lncRNA *Cyrano*, two miRNAs miR-7 and miR-671, and the circRNA *Cdr1as* (**Box 2**). Because *Cdr1as* contains multiple binding sites for miR-7, some of them with quite extensive complementarity, it is puzzling to observe that *Cdr1as* does not trigger miR-7 decay through TDMD. In fact, *Cdr1as* depletion in KO mice leads to a reduction in miR-7 through an unknown mechanism (Piwecka et al., 2017). On the other hand, *Cdr1as* full depletion or disruption of its miR-671 site causes a small increase (∼1.8-fold) in miR-671 levels, supporting the idea that *Cdr1as* might direct some degradation of miR-671 through TDMD (Piwecka et al., 2017; Kleaveland et al., 2018). These observations challenge our knowledge of TDMD and await further clarification regarding the target RNA nature and their effects on miRNA stability.

Box 2 The Cyrano•miR-7 family•Cdr1as•miR-671 regulatory network. The most highly conserved region of *Cyrano* contains a site with high (“TDMD-competent”) complementarity to the *miR-7* family, expressed from three loci that encode two highly similar miRNA variants, miR-7a and miR-7b. *Cdr1as* is a circRNA conserved in mammals with a role in neuronal activity (Piwecka et al., 2017). *Cdr1as* contains multiples sites (130 in mouse and 73 in human) for miR-7, as well as a miR-671 site with sufficient complementarity to allow slicing by AGO2. Genetic studies using combinations of gene disruptions and mutations revealed several interactions between these RNAs: (1) *Cyrano* induces degradation of miR-7 through TDMD; (2) miR-7 reduces *Cdr1as* levels in part via stimulation of its slicing by miR-671 and also through an unknown, possibly indirect, mechanism; (3) *Cdr1as* appears to cause a modest TDMD of miR-671; (4) with its unusually high number of sites, *Cdr1as* is capable of binding a huge number molecules of miR-7 and was initially proposed to function as a sponge (Hansen et al., 2013; Memczak et al., 2013); (5) alternatively, *Cdr1as* might protect miR-7 from degradation (Piwecka et al., 2017). In this scenario, because miR-671 can trigger its destruction, *Cdr1as* could transport miR-7 and/or RBPs to subcellular locations where miR-671 could prompt the release of its cargo (Memczak et al., 2013). In *Cyrano*-deficient mice, the levels of both miR-7a and 7b were greatly increased in several, but not all, tested tissues, as a result of impaired degradation of miR-7 by *Cyrano* through TDMD. This effect was more prominent in brain tissues and depended on the basal miR-7-*Cyrano* expression stoichiometry: the greater the expression of *Cyrano* and the lower the expression of miR-7 in a given tissue, the greater the increase of miR-7 levels following *Cyrano* KO. Increased miR-7 levels in turn reduced *Cdr1as* levels throughout the brain, in a manner that correlated with the magnitude of miR-7 variation (Kleaveland et al., 2018). Additionally, the increase in miR-7 correlated with a modest but significant enhanced repression of miR-7 predicted targets in some, though not all, tissues in *Cyrano^-/-^* mice: the effect was more prominent in tissues with the highest basal expression of miR-7, while tissues with the lowest expression levels of miR-7 showed no effect on miR-7 targets—in spite of their largest increase in miR-7 upon Cyrano depletion. In this way, both *Cyrano* and *Cdr1as*, together with miR-7 and miR-671, constitute a ncRNA regulatory network physically and functionally localized throughout the cytoplasm, in which TDMD participates as a key player. The biological benefit of simultaneously producing and efficiently degrading both miR-7 family members in some tissues remains an open question. Perhaps this creates the conditions to add dynamism to miR-7 regulation within the appropriate spatial and temporal requirements in specific cell types, rather than globally altering miR-7 levels to ultimately affect downstream targets in steady state. This appears nicely correlated with the high potency of TDMD observed in neuronal cells, where much of their post-transcriptional regulation involves fast and localized regulation of miRNAs and their targets (Rajasethupathy et al., 2009; Sethi and Lukiw, 2009; Krol et al., 2010a; Sambandan et al., 2017) (reviews by Holt and Schuman, 2013; Glock et al., 2017) and where TDMD could play an important role (see main text).

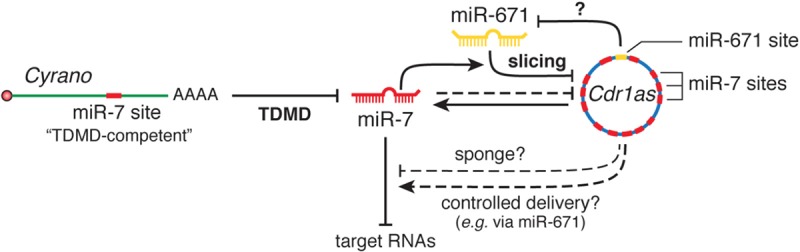

TDMD seems to be particularly efficient in neuronal cells (see below), where most known endogenous examples of TDMD have been discovered so far. However, a very recent report has identified several hundreds of target RNAs with potential TDMD activity in mouse fibroblasts (Ghini et al., 2018). In particular, the study demonstrated that the endogenous mRNA *Serpine1* controls the degradation of two miRNAs, miR-30b-5p and miR-30c-5p. Upon serum stimulation, upregulation of *Serpine1* mRNA reduces miR-30b/c levels through TDMD and therefore limits their activity toward other targets, resulting in the modulation of gene expression and cellular phenotypes such as cell cycle re-entry and apoptosis.

The number of additional endogenous target RNAs naturally triggering TDMD is likely to be limited. This is because the types of non-canonical miRNA binding sites that present extensive pairing to their targets are infrequent. For example 3′-supplementary and 3′-compensatory sites, featuring different degrees of pairing to the miRNA seed and 3′ regions, account for about 5% and 1%, respectively, of the preferentially conserved miRNA sites in mammalian mRNAs (Friedman et al., 2009). Yet, there still might be more TDMD events than possibly anticipated due to several reasons. First, mRNAs with non-conserved sites are repressed by miRNAs as often as mRNAs with conserved sites (Krutzfeldt et al., 2005; Lim et al., 2005; Giraldez et al., 2006; Rodriguez et al., 2007), thus a similar scenario is conceivable for TDMD (i.e., species-specific instances of TDMD might be discovered). Second, we are likely lacking the full spectrum of miRNA–target RNAs architectures that can trigger TDMD (see below). Finally, our ability to predict miRNA binding sites on target RNAs is limited (**Box 3**). Therefore, getting to know the actual abundance of endogenous target RNAs triggering TDMD needs further investigation.

**Box 3. Challenges in miRNA target prediction.**

The search of miRNA–target RNA interactions and the prediction of the fate of each molecule (either target RNA turnover or miRNA degradation by TDMD) is hindered by the lack of effective sequence-based predictive computational tools. These algorithms, based on sequence evolutionary conservation (Targetscan) (Lewis et al., 2005; Agarwal et al., 2015) or on sequence specificity rules (RNA22, PITA) (Miranda et al., 2006; Kertesz et al., 2007), still generate prediction sets with high false positive rates (Pinzon et al., 2017). The fact that miRNA–RNA recognition rules in different biological contexts are still not fully understood and that the outcome of those interactions depends on the concentration of the interacting molecules (usually not known before experimental measurements take place in any specific experimental setup) result in poor prediction power. Other phenomena such as the specific titration of miRNAs by RNA targets, without any effect on the concentration of the involved molecules, also take place, adding an extra layer of complexity to the study of these interactions (Seitz, 2009; Pinzon et al., 2017).

The lack of consistency of algorithm-based predictions has propelled the efforts to generate experimental miRNA–RNA interaction maps using biochemical and sequencing based techniques including CLIP-Seq/HITS-CLIP (Ule et al., 2005; Licatalosi et al., 2008), PAR-CLIP (Hafner et al., 2010) or CLASH (Helwak et al., 2013) (reviewed in Hausser and Zavolan, 2014). These techniques, developed to map RNA–protein and RNA–RNA interactions, have been used to chart the transcriptome-wide binding sites of miRNA containing ribonucleoprotein complexes. In order to map miRNA interactors into the transcriptome, these techniques rely on the crosslinking, immunoprecipitation, and sequencing of the RNA molecules bound to the RISC complex (CLIP-Seq/HITS-CLIP, PAR-CLIP) (Chi et al., 2009; Hafner et al., 2010). Certain techniques incorporate an extra step to ligate miRNAs to their interacting RNAs producing “RNA chimeras” that can be sequenced and identified (Helwak et al., 2013; Moore et al., 2015). Novel bioinformatic tools now allow to search for miRNA–RNA interactions based on the experimental datasets generated using these transcriptome-wide techniques, including StarBase (Yang et al., 2011) and doRiNA (Blin et al., 2015). Nevertheless, the very nature of TDMD (which implies the loss of miRNAs) makes the identification of target RNAs engaged in this phenomenon difficult to detect and measure in steady state experimental conditions. For instance, as for canonical miRNA target capturing (Hausser and Zavolan, 2014), in situations where the rate of target dissociation from RISC is faster than the miRNA decay rate via TDMD, the data based on biochemical and sequencing methods would allow to identify potential TDMD-competent sites. On the contrary, situations where TDMD occurs faster than target dissociation from RISC would preclude identification of high affinity TDMD-competent sites from this type of experimental approaches. The ultimate strategy to identify and investigate TDMD-competent targets is still the measurement of the concentration of miRNAs under the disruption of the interaction with the known RNA interactor. This can be achieved, for example, using mutant reporters or disrupting genomic interaction sites using the current genome editing technologies. In the future, once the enzymes involved in TDMD are identified, CLIP-Seq-like techniques will be useful to map potential genome-wide TDMD specific miRNA–RNA interactions.

## Mechanism of TDMD

### miRNA–Target Architecture

Despite the different experimental systems used to study TDMD, clear similarities have emerged between them in relation to the miRNA–target architectures that effectively enter the pathway. Both in flies and in mammals, a more extensive complementarity than the one typically found between miRNAs and their targets is required to trigger TDMD, while target sites that resemble canonical natural miRNA binding sites—i.e., mostly sites paired to the miRNA seed region only or to both the seed and a small 3′ supplementary pairing—do not (Ameres et al., 2010; Xie et al., 2012; de la Mata et al., 2015) (**Figure 1**). When assayed *in vitro* using fly lysates, targets with a seed match and up to 8 mismatches within the 3′ end of the miRNA are enough to trigger TDMD. A small central bulge between 3 and 7 nt, flanked by fully complementary sequence, also triggers TDMD in flies (*in vitro*) and in mammals (in cell culture), respectively. Additionally, target RNAs with a central bulge of 4 nt allow up to two additional mismatches within the 3′ end of the miRNA to trigger a significant TDMD response in mammalian neurons (de la Mata et al., 2015). Through an architecture that fits these criteria, MCMV’s highly complementary single site within the *m169* transcript is sufficient to trigger an effective miRNA degradation, making the additional hundreds of potential miR-27 targets throughout its transcriptome dispensable for regulation (Libri et al., 2012). Yet, *m169*’s site for miR-27 does not tolerate even a single mismatch at the miRNA 3′ end in order to maintain TDMD, probably due to the large central bulge present at this site (Haas et al., 2016). Indeed, although overall similar in miR-27 binding site architecture, MCMV’s *m169* site features a slightly larger bulge than in *H. saimiri HSUR-1* transcript plus three G-U (wobble) base pairs. Thus, the amount of unpaired bases tolerated for productive TDMD might be different for different cell types or conditions and larger than currently estimated (Ameres et al., 2010; de la Mata et al., 2015), and questions as to what are the minimal and more optimal TDMD-provoking site architectures have not been fully answered yet. For instance, compared to other architectures, asymmetrical bulges significantly increase the efficacy of miRNA decoys (Haraguchi et al., 2009) (**Figure 2A**). Perhaps, structural differences like the ones described above could explain why the asymmetrically bulged miR-17 and miR-20a binding sites within HCMV’s *UL144-145* target mRNA trigger a potent TDMD despite containing three mismatches within the 3′ end of the miRNA, which would otherwise lead to unproductive TDMD (de la Mata et al., 2015).

As for canonical miRNA silencing of its targets, TDMD requires pairing to the seed of miRNAs (Ameres et al., 2010; Marcinowski et al., 2012; Lee et al., 2013; de la Mata et al., 2015). Consistently, base-pairing between MCMV *m169* and miR-27 seed is needed for maximal miRNA degradation. However, although to a low extent, an *m169* version carrying a point mutation in the seed sequence partially triggered TDMD (Marcinowski et al., 2012). Likewise, “seedless” miRNA binding sites within artificial target RNAs have been shown to elicit TDMD *in vitro* (Park et al., 2017) (**Figure 2B**). This mode of non-seed base-pairing has been shown to be relatively prevalent according to ligation-based biochemical analysis, and seems to mediate a modest repression on its targets (Helwak et al., 2013). Yet it remains to be demonstrated whether the presumably unfavorable thermodynamic properties of such miRNA–target interactions (Haley and Zamore, 2004; Wee et al., 2012) are truly capable of inducing TDMD within endogenous settings. From these considerations it emerges that the binding architecture requirements for TDMD might differ for different miRNAs and in different systems. Also, in addition to the influence of thermodynamics and kinetics on miRNA–target interaction during TDMD, it is conceivable that high expression levels of targets might pay the penalty of a lower degree of complementarity, especially in situations of limited seed pairing, as shown for canonical miRNA silencing (Brancati and Grosshans, 2018).

In addition to the highly complementary binding required for TDMD, proximal auxiliary sequences can boost its activity, as described for the 3′ additional motif in UL144-145 mRNA (Lee et al., 2013). Similarly, the *HSUR-1* miR-27-binding region must be available in a conformationally flexible region for TDMD to be active, being evidenced by the fact that *HSUR-1* mutants with an intact miR-27 binding site that is artificially sequestered in predicted helices lose their ability to trigger TDMD (Pawlica et al., 2016). The same may be true for more distal sequences within endogenous transcripts. For instance, it has been speculated that multiple conserved regions within *Cyrano* lncRNA might boost its TDMD efficacy (Kleaveland et al., 2018). On the other hand, the pressure to keep viral genome size small might have favored viruses to use a larger number of shorter and less effective transcripts. In spite of the current progress, answers await to determine whether differences in TDMD efficacy are mainly due to sequence constraints of the inducing target RNA or rather to cell-type specific properties.

### Tailing, Trimming, and Exonucleolytic Degradation of miRNAs

For the many artificial and natural targets described above, a common theme during TDMD is that miRNA degradation strongly correlates with tailing and trimming at the 3′ ends of the miRNAs, which has fostered the idea that tailed species might represent actual intermediates for subsequent degradation by 3′-to-5′ exonucleases (**Figure 1**). Tailing results from the addition of non-templated nucleotides by terminal nucleotidyl transferases (TNTases), which gives rise to 3-end diversity in several types of RNAs including miRNAs. TNTases belong to the DNA polymerase-β superfamily that includes poly(A) polymerase, responsible for the addition of poly(A) tails to mRNAs, and up to 12 TNTases have been described in humans (Martin and Keller, 2007). Individual members of this family of enzymes display a notable substrate flexibility and have been implicated in modification of substrates belonging to distinct classes of coding and/or non-coding cellular RNAs (Martin and Keller, 2007). Most commonly, TNTase’s activity incorporates adenosine or uridine *in vivo* (Norbury, 2013), and examples exist of single enzymes displaying both activities on different substrates (Trippe et al., 1998; Mellman et al., 2008). Modification of RNA 3′ ends by TNTases plays a versatile role on the modified RNAs and has been shown to affect the maturation, function, and turnover of coding and ncRNAs (Norbury, 2013; Scott and Norbury, 2013). For instance, while poly(A) tails trigger mRNA decay in bacteria, they enhance mRNA stability and translation in eukaryotes (Dreyfus and Regnier, 2002). Similarly, TNTases have been implicated in the regulation of precursor and mature miRNAs, eliciting sometimes opposite effects on different miRNAs and in different cell systems (**Table 1**). In support of the idea that tailed miRNA species might represent degradation intermediates, it is known that tailing of certain metazoan pre-miRNAs (Chang et al., 2013) and mature miRNAs (Boele et al., 2014; Katoh et al., 2015) leads to their degradation. Similar TNTase activities have been described in flies and plants, where uridylation of miRNAs can lead to their degradation (**Table 1** and see below) and points at tailing as a conserved mechanism that regulates miRNA turnover. To date, only one TNTase, TUT1, has been shown to copurify with TDMD nucleoprotein complexes (together with DIS3L2 exoribonuclease, see below) in HeLa cells. However, knockdown of TUT1 does not affect TDMD efficiency, probably due to redundancy with other TNTases (Haas et al., 2016).

**Table 1:** TNTases affecting miRNA 3′ ends.

Factor	Reported activity	Effect on pre- or miRNA	Reference
ZCCHC11 (TUT4)	Oligouridylates *pre-let-7* (by complexing with the pluripotency factor LIN28)	Oligo(U) tail prevents efficient substrate recognition by Dicer, and functions as a decay signal for the Perlman syndrome exonuclease DIS3L2	Hagan et al., 2009; Heo et al., 2009; Chang et al., 2013
	Uridylates mature, single-stranded miRNAs, including miR-26 family members (1–3 uridine additions)	Abrogates repression by miR-26 on interleukin-6	Jones et al., 2009
	Uridylates a small subset of miRNAs (let-7, miR-99/100, miR-196a/b, and miR-10a/b family members) (similar as ZCCHC6)	miRNA stability not affected	Thornton et al., 2014
	Monoadenylates miR-31	Not determined	Wyman et al., 2011
ZCCHC6 (TUT7)	In the absence of LIN28 (and together with ZCCHC11), it monouridylates class II pre-miRNAs, including members of the *pre-let-7* family	Mono uridylation enhances processing by Dicer: it restores the 2-nt 3′ overhang of pre-miRNAs that are imprecisely cleavage by Drosha	Heo et al., 2012
	Uridylates a small subset of miRNAs (let-7, miR-99/100, miR-196a/b, and miR-10a/b family members) (similar as ZCCHC11)	miRNA stability not affected	Thornton et al., 2014
	Monouridylates let-7e	Not determined	Wyman et al., 2011
GLD2 (TUT2/PAPD4)	Monoadenylates mature miR-122. (miR-122 derives from the 5′ arm of the pre-miRNA, i.e., GLD2 must act on the mature miRNA after dicing)	Enhances mature miR-122 stability	Katoh et al., 2009; Burns et al., 2011
	Oligoadenylates mature miR-122 (perhaps involving a yet-unknown adaptor protein, such as in the case of ZCCHC11, whose processivity is enhanced by LIN28)	Promotes degradation of miR-122 through PARN	Katoh et al., 2015
	Monoadenylates specific miRNAs	Enhances stability of a subset of mature miRNA, though not globally	Burroughs et al., 2010; Wyman et al., 2011; D’Ambrogio et al., 2012; Mansur et al., 2016
PAPD5 (TUT3)	Oligoadenylates miR-21 [non-canonical poly(A) polymerase]	Enhances miR-21 degradation by PARN	Boele et al., 2014
	Monoadenylates specific miRNAs	Not determined	Wyman et al., 2011
TUT1 (PAPD2)	Interacts with tailed and trimmed isoforms of miR-27, particularly upon TDMD induction	Does not inhibit TDMD when downregulated, likely due to redundancy with other TNTases	Haas et al., 2016
	Monouridylates miR-200a and monoadenylates miR-31	Not determined	Wyman et al., 2011
MTAP (PAPD1)	Monoadenylates miR-106b (also promotes miR-1246 3′ GA addition)	Not determined	Wyman et al., 2011
HESO1 (HEN1 SUPPRESSOR 1) Plant enzyme (*A. thaliana*)	Oligouridylates unmethylated small RNAs (miRNAs and siRNAs). Prefers U-ending miRNAs as substrates.	Triggers miRNA decay	Li et al., 2005; Yu et al., 2005; Ren et al., 2012; Zhao et al., 2012
URT1 Plant enzyme (*A. thaliana*)	Uridylates (likely single U additions) unmethylated miRNAs. Prefers A-ending miRNAs as substrates.	Triggers miRNA decay Reduces slicer activity when uridylating miR165/6 *in vitro*	Tu et al., 2015; Wang et al., 2015
Unknown factor (*A. thaliana*)	Adenylates specific miRNAs	Seems to enhance miRNA stability	Lu et al., 2009
MUT68 Green alga *Chlamydomonas reinhardtii*	Oligouridylates and oligoadenylates unmethylated small RNAs (miRNAs and siRNAs)	Triggers miRNA decay by the catalytic exosome subunit RRP6	Ibrahim et al., 2010


Different exoribonucleases are known to degrade miRNAs in different organisms, both in the 5′-to-3′ and 3′-to-5′ direction, with the latter being a more widespread phenomenon (**Table 2**) (reviewed in Ruegger and Grosshans, 2012). During TDMD, the appearance of 3′-end trimmed species suggests that degradation might occur from this end of miRNAs. Indeed, several 3′-to-5′ exonucleases have been shown to degrade miRNAs, although it remains unclear whether specific ones are associated with the process of TDMD. For instance, PARN can preferentially degrade 3′-tailed miRNAs (Boele et al., 2014; Katoh et al., 2015), though this activity has not been shown to occur in response to miRNAs pairing to targets. At present, DIS3L2 is the only exoribonuclease shown to catalyze miRNA degradation upon binding to a highly complementary target (Haas et al., 2016). DIS3L2 belongs to the RNase II/R family of exoribonucleases and is conserved in fission yeast, plants (aka SOV), and animals (Gallouzi and Wilusz, 2013). This enzyme belongs to the DIS3 family but unlike its two other homologs, DIS3 and DIS3L, it is not associated with the RNA exosome (Lubas et al., 2013; Malecki et al., 2013). DIS3L2 copurifies with TDMD nucleoprotein complexes when transfecting an antimiR-27 (2′-*O*-methylated and biotinylated) oligoribonucleotide mimicking the *m169* transcript in HeLa cells. Interestingly, the complex includes TUT1, exoribonuclease XRN2, and RNA-induced silencing complex (RISC) factors such as AGO1, 2 and 3, TNRC6B and RBM4. Furthermore, both depletion of endogenous DIS3L2 and overexpression of a catalytic mutant of DIS3L2—with dominant negative effect—partially impairs miR-27 trimming induced by viral *m169* target RNA (Haas et al., 2016). Unlike m169, *Cyrano*-triggered TDMD seems to be independent of DIS3L2 (Kleaveland et al., 2018). However, since DIS3L2 has a preference for uridylated targets (Gallouzi and Wilusz, 2013) (**Table 2**), and because *m169* target RNA mainly induces uridine additions, while *Cyrano* induces adenine additions on their bound miRNAs (see below), it is conceivable that DIS3L2 mediates miRNA degradation during TDMD in cases where targets preferentially induce uridylation rather than adenylation on their cognate miRNA 3′ ends. At any rate, it remains to be determined which additional nucleases are responsible for miRNA degradation in other described cases of TDMD.

**Table 2:** Nucleases involved in miRNA degradation.

Factor	Reported activity	Effect on pre- or miRNA	Reference
SDN family (small RNA degrading nuclease) Plant enzyme (*A. thaliana*)	3′-to-5′ exoribonucleases	Degrades miRNAs (and siRNAs) (although unable to degrade uridylated miRNAs)	Ramachandran and Chen, 2008
Exosome complex (Rrp41)	3′-to-5′ exoribonuclease	Degrades miR-382 (direct or indirect effect remains to be determined)	Bail et al., 2010
XRN-1	5′-to-3′ exoribonuclease	Degrades miR-382, though to a lower extent than the exosome (direct or indirect degradation of miR-382 remains to be determined)	Bail et al., 2010
XRN-2 *(C. elegans)*	5′-to-3′ exoribonuclease	Degrades let-7 and other mature miRNAs in *Caenorhabditis elegans*.	Chatterjee and Grosshans, 2009
PNPase*^old-3^*	3′-to-5′ exoribonuclease	Degrades specific mature miRNAs (miR-221, miR-222, and miR-106b)	Das et al., 2010
PARN	3′-to-5′ exoribonuclease	Degrades miR-21Degrades miR-122	[Bibr B18][Bibr B95]
DIS3L2 (Perlman syndrome exonuclease)	3′-to-5′ exoribonuclease	Degrades oligouridylated pre-let-7 Degrades miR-27 during virus-induced TDMD	Chang et al., 2013; Ustianenko et al., 2013; Faehnle et al., 2014 Haas et al., 2016


**FIGURE 3:**
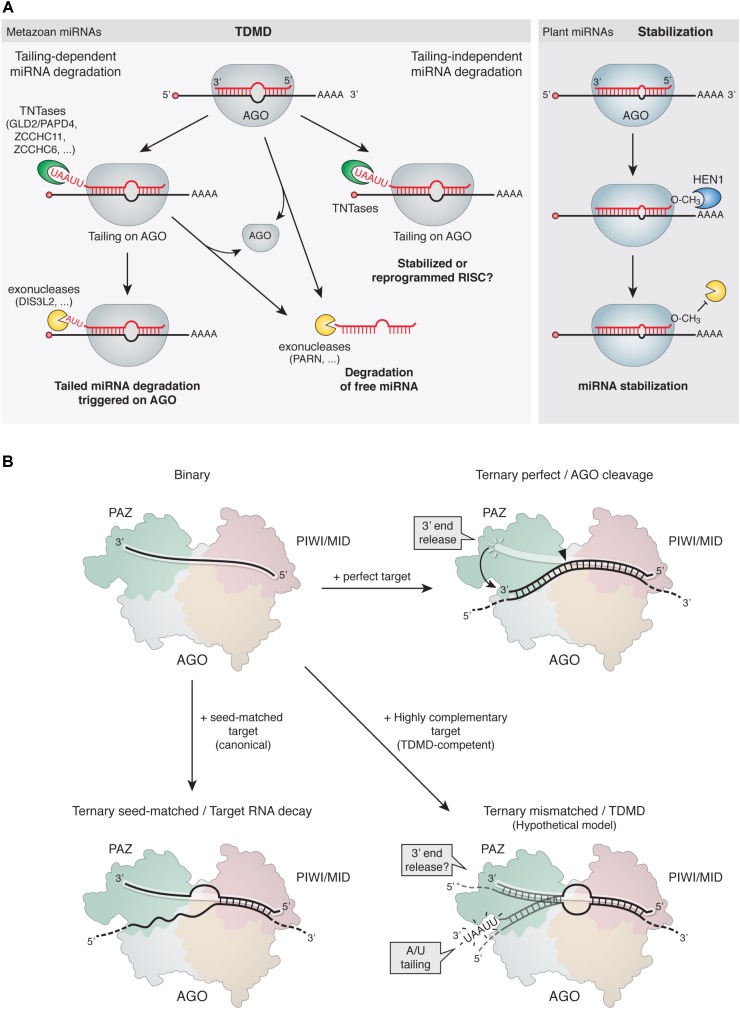
Mechanism of TDMD. **(A,** Left) Proposed models for TDMD. In tailing-dependent miRNA degradation, highly complementary target RNAs would either expose the miRNA 3′ ends to TNTases by detaching them from AGO’s 3′-end binding pocket in the PAZ domain (see panel **B**), favor the kinetics of TNTases on the 3′ ends of the bound miRNAs, or both. The tailed species would subsequently serve as preferred substrates for 3′-to-5′ exoribonucleases. Alternatively, tailing might promote miRNA unloading from AGO for subsequent degradation of free miRNAs in the cytoplasm. In tailing-independent miRNA degradation, highly complementary target RNA would promote unloading of miRNAs from AGO, leading to subsequent degradation of free miRNAs in the cytoplasm. Increased tailing could be a parallel process, rather than a cause of degradation, induced by highly complementary target RNAs binding to miRNAs. Tailing could in turn have either stabilizing effects or an influence on the activity of miRNAs. (Right) In plants, 2′-*O*-methylation at the 3′ end of miRNAs prevents 3′ tailing and stabilizes the modified miRNAs (see main text). **(B)** Structural rearrangements induced on AGO by binding of target RNAs with different architectures. (Top left) Binary complex of a miRNA loaded on AGO; the miRNA 5′ end is bound to the MID domain while the 3′ end is bound to the PAZ domain and therefore protected from terminal modifications. (Top right) Ternary complex comprising an AGO-loaded siRNA (or miRNA) bound to a perfectly complementary target RNA; the extensive 3′ pairing releases the 3′ end of the siRNA and induces a conformational change that switches the complex from an inactive to an active slicing conformation, cleaving the target at the catalytic site (solid arrowhead). The rapid release of the cleaved target would preclude tailing of the AGO-bound miRNA, which would return to its protected conformation. (Bottom left) Ternary complex comprising an AGO-loaded miRNA bound to a canonical seed-matched target RNA. Due to lack of pairing to the 3′ end of the miRNA, the 3′ end would remain bound and protected within the AGO PAZ domain. (Bottom right) Hypothetical structure of a ternary complex comprising an AGO-loaded miRNA bound to a TDMD-competent target RNA; the extensive 3′ pairing might expose the 3′ end in a conformation that would render it susceptible to attack by modifying enzymes (e.g., TNTases).

In addition to the above evidence, certain TNTases and 3′-to-5′ exoribonucleases have been shown to interact and act together in protein complexes (Kim and Richter, 2006; Reimao-Pinto et al., 2016), which is further consistent with the idea that tailed species act as degradation intermediates during TDMD. The fact that highly complementary RNA targets induce 3′ tailing while miRNAs are still loaded on AGO, suggests that the degradation machinery can act in close proximity to, or in association with, RISC (de la Mata et al., 2015; Haas et al., 2016) (**Figure 3A**). This is reminiscent of the case in plants, where uridylation occurs on AGO-bound miRNAs (see below). Moreover, evidence shows that the mature (untailed) miRNA isoform is more extensively depleted from the AGO-associated than from the total RNA pool (de la Mata et al., 2015). Hence, it seems possible that tailing of AGO-bound miRNAs is the first step in a pathway that ultimately leads to degradation and/or ejection of the miRNA from the RISC complex.

### Lessons From Plant miRNAs and Other Metazoan Small RNAs

Different classes of small regulatory RNAs such as plant miRNAs and siRNAs, fly siRNA and metazoan piRNAs (piwi-interacting RNA), feature an extensive base pairing to most of their targets. In plants, miRNAs and their target mRNAs have nearly perfect complementarity and, consequently, transcript cleavage by plant miRNAs is frequent (Chen, 2005; Jones-Rhoades et al., 2006). From our knowledge of TDMD, one could expect these small RNA species to be occasionally destabilized by such interactions. However, these small RNAs are also typically modified at their 3′ ends by a stabilizing 2′-*O*-methylation which is introduced by homologs of the plant methyltransferase HEN1 (Hua enhancer 1) (Yu et al., 2005; Yang et al., 2006). HEN1 acts either at a double stranded or single stranded stage depending on the class of RNA and organism species (Vagin et al., 2006; Horwich et al., 2007; Houwing et al., 2007; Kirino and Mourelatos, 2007a,b; Saito et al., 2007; Kamminga et al., 2010) (reviewed in Ji and Chen, 2012). For example, plant HEN1 methylates both siRNA and miRNA at the dsRNA stage before they are loaded into AGO, while in flies, and likely in all arthropods, HEN1 methylates siRNAs at the last step in Ago2-RISC assembly. 2′-*O*-methylation typically prevents 3′ tailing and stabilizes the modified small RNAs (**Figure 3A**). In fact, *hen1* mutants lead to both 3′ truncation and 3′ uridylation of miRNAs or piRNAs in plants (Li et al., 2005; Abe et al., 2010; Zhai et al., 2013), *Drosophila* (Horwich et al., 2007; Saito et al., 2007), *C. elegans* (Billi et al., 2012), zebrafish (Kamminga et al., 2010), and mouse (Kirino and Mourelatos, 2007b). In *Arabidopsis hen1* mutant, the TNTases HESO1 and URT1 uridylate unmethylated miRNAs with different substrate specificities (**Table 2**), leading to miRNA degradation (reviewed in Yu et al., 2017b). Yet, the exoribonuclease/s responsible for degrading uridylated miRNAs in plants have so far not been described. For instance, the *Arabidopsis* orthologs of DIS3L2 (Suppressor of Varicose, SOV) and exosome subunit RRP6 have no known role in degrading uridylated miRNAs. Instead, SDN family of nucleases are among the few exoribonucleases known to degrade specific miRNAs in plants, although they are unable to degrade uridylated miRNAs while they are active against methylated miRNAs (Ramachandran and Chen, 2008; Yu et al., 2017a). Interestingly, both SDN1 and HESO1/URT1 have been shown to act on miRNAs which are bound to AGO1 (Zhai et al., 2013), partly due to HESO1/URT1-AGO1 direct interaction (Tu et al., 2015; Wang et al., 2015). This has led to the hypothesis that SDN1 and HESO1/URT1 cooperate, with SDN1 removing the methyl group from these miRNAs and HESO1/URT1 triggering a subsequent uridylation that ultimately leads to miRNA degradation by a yet-to-be-determined exonuclease (Yu et al., 2017b). Together, this evidence is consistent with a model of tailing as an intermediate step in miRNA degradation, which might operate in miRNA degradation triggered by highly complementary targets during TDMD.

Despite HEN1’s protection of plant miRNAs, targets can have an impact on their activity and conceivably on their stability. This idea first emerged from the concept of “target mimicry” in plants, where a target RNA with a bulged site preventing cleavage by miR-399, instead sequestered the miRNA and inhibited its activity (Franco-Zorrilla et al., 2007).

### Tailing and Trimming Independence of miRNA Degradation

Despite the association between tailing and miRNA turnover, it still remains unclear whether tailed and trimmed miRNA species are *bona fide* intermediates of miRNA degradation during TDMD or rather the consequence of independent parallel processes. Accordingly, miRNA degradation induced by *Cyrano* is associated with, but apparently independent on, tailing and trimming of the miRNA 3′ end. Unlike most cases in mammals where both adenylation and uridylation have been reported (Ameres et al., 2010; Baccarini et al., 2011; Marcinowski et al., 2012; Xie et al., 2012; de la Mata et al., 2015), *Cyrano* mostly induces the addition of 2 or more untemplated adenosines rather than uridines, with little effect on monoadenylation and tailing with other nucleotides (Kleaveland et al., 2018). This tailing pattern recapitulates the one induced by the cytoplasmic poly(A) polymerase GLD2. However, despite the potential role of GLD2 in mediating tailing induced by *Cyrano*, and consistent with previous reports (Mansur et al., 2016), loss of GLD2 prevents A-tailing but causes no change in the proportion of trimmed miR-7 species, neither does it affect the levels of miR-7. As a consequence, this could imply that adenylation and degradation of miR-7 induced by *Cyrano* are uncoupled, and, even more globally, that tailing is not an intermediate step of TDMD (Kleaveland et al., 2018). Although it will be instructive to determine whether additional, probably less abundant—in steady state—3′ end additions added by redundant TNTases also participate in this regulation, these results raise the question of what could be their role during tailing-independent decay induced by targets. Perhaps tailed miRNA species accumulating upon binding to highly complementary target RNAs represent stabilized or even reprogrammed RISC complexes (**Figure 3A**). At any rate, highly complementary target RNAs would appear to play a dual role by independently inducing tailing and unloading of miRNAs from AGO. Since AGO–miRNA complexes are thought to be extremely stable, targets with TDMD-competent architectures could promote an efficient release of miRNAs from RISC and expose them for degradation by nucleases (**Figure 3A**). This has been suggested by a report showing that guide RNAs within AGO2 can be dissociated easily upon binding to a highly complementary target RNA *in vitro* (De et al., 2013). Degradation of the free miRNA after release from RISC could then occur by redundant nucleases. For instance, PARN-mediated degradation of miR-122 loaded onto AGO2 *in vitro* is markedly lower than PARN-mediated degradation of free miR-122, suggesting that PARN-mediated miRNA degradation takes place outside RISC (Katoh et al., 2015), while AGO2 protects miRNAs from degradation by PARN. In this sense, the degradation of miRNAs outside RISCs seems similar to the XRN-1/XRN-2- mediated degradation of miRNAs in *C. elegans* (Chatterjee and Grosshans, 2009; Bosse et al., 2013).

Determining whether tailed miRNA species actually represent degradation intermediates faces a number of challenges. First, different TNTases are known to add 3′ nucleotides in a miRNA- and developmental stage-specific manner, and often do not seem to affect miRNA stability (Wyman et al., 2011; Thornton et al., 2014). For instance, ZCCHC6 (TUT7) and ZCCHC11 (TUT4) expression is developmentally regulated and both can mono-uridylate the 3′ ends of a specific subset of miRNAs [involved in cell differentiation and Homeobox (Hox) gene control] without affecting miRNA abundance in cultured cells, not even of those miRNAs predicted as preferred substrates of these two TNTases (Thornton et al., 2014). Second, the differential activity of individual TNTases might have opposite effects depending on the length of the introduced tail. Indeed, monoadenylation by GLD2 stabilizes miR-122 (Katoh et al., 2009; Burns et al., 2011), whereas oligoadenylation by GLD2—perhaps involving an adaptor protein—promotes degradation of miR-122 by enhancing PARN-mediated exonucleolytic decay (Katoh et al., 2015) (**Table 1**). Third, TNTases exhibit redundancy, and compensatory effects can operate among them. In fact, knockdown of individual TNTases is often associated with increased expression of other TNTase family members, raising the possibility that functional compensation by one enzyme for another may occur in some cases (Wyman et al., 2011; Thornton et al., 2014). This may in turn blur the knockdown effects of individual or combined TNTases on 3′ tailing. Fourth, so far most studies have examined only the steady state levels of 3′ tailed species, thus the mechanisms by which each enzyme modulates the observed miRNA 3′ nucleotide addition frequency—either via altered kinetics of 3′ additions or effects on miRNA degradation—remain to be determined. Therefore, the role of tailing as a potential intermediate modification en route to miRNA degradation during TDMD remains an open question that requires further investigation.

### miRNA Decay vs. Target Decay

Because the degradation of 5′ cleavage products of target RNAs in diverse organisms involves 3′ end tailing downstream of the cleavage site, it is conceivable that enzymes that can tail both miRNAs or their targets are recruited when AGO proteins bind to an RNA (Shen and Goodman, 2004; Ibrahim et al., 2006), potentially involving a similar mechanism and even co-degradation of both RNA species. However, mammalian miRNAs only rarely bear extensive enough pairing to trigger slicing by AGO2, and mechanisms that lead to miRNA–mediated target RNA decay typically follow a different pathway (reviewed in Jonas and Izaurralde, 2015 and Bartel, 2018). In fact, existing evidence argues against a model where miRNAs and their targets are co-degraded during TDMD. Instead, TDMD and target degradation appear to be two independent and competing processes, whose balance can be shifted by alterations in miRNA and target relative abundances (de la Mata et al., 2015). Moreover, typical mRNA degradation induced by miRNAs (Grimson et al., 2007; Saetrom et al., 2007; Broderick et al., 2011), but not miRNA degradation induced by TDMD (de la Mata et al., 2015; Haas et al., 2016), relies on cooperativity among multiple target sites to reach high efficacy. This is evidenced by the ability of individual target mRNA molecules to induce decay of multiple miRNA molecules independently of the number of binding sites present. In this way, TDMD might be able to regulate miRNAs without requiring as high target RNAs levels as other previously described mechanisms (see below).

### TDMD vs. Competition by Pseudo-Targets/ceRNAs

Current prediction strategies relying on both high-throughput biochemical and computational approaches (**Box 3**) predict that each miRNA might regulate tens to hundreds of genes (Friedman et al., 2009). Yet many of those sites seem to mediate little to no repression by miRNAs, and frequently appear as false positive target predictions (Pinzon et al., 2017). Intriguingly, despite their lack of repression, they still display phylogenetic conservation. Various reasons, including neutral evolution (Ameres and Zamore, 2013), may account for this apparently puzzling observation. Among the probable causes, it has been suggested that some of those conserved sites might have been selected for titrating rather than for being regulated by miRNAs (Seitz, 2009; Pinzon et al., 2017). The occurrence of this so called “pseudo-targets” has been demonstrated for a few highly abundant mRNAs (Pinzon et al., 2017). A similar concept has been proposed based on the idea that individual endogenous transcripts containing miRNA binding sites can act as competing endogenous RNAs (ceRNAs) and regulate other miRNA binding site-containing transcripts through increasing or decreasing the miRNA activity (Poliseno et al., 2010; Cesana et al., 2011; Salmena et al., 2011). However, more recent studies have questioned the likelihood of the ceRNA hypothesis by showing that an efficient titration of miRNAs by individual mRNAs must be rare *in vivo*, usually requiring exceptionally high target concentrations (Bosson et al., 2014; Denzler et al., 2014, 2016). Unlike competition by pseudo-targets or ceRNAs, TDMD likely involves a multiple turnover process that allows the destruction or several copies of miRNA molecules per target RNA (de la Mata et al., 2015; Kleaveland et al., 2018). For instance, *Cyrano*-mediated TDMD exhibits a very high efficiency in neurons, such that proportionally higher molar concentrations of miR-7 are significantly degraded by substoichiometric levels of *Cyrano*. Therefore, by relying on enzymatic activity, TDMD-inducing targets differentiate from the titrating activity of pseudo-targets or ceRNAs in that TDMD requires relatively lower target RNA concentrations to achieve significant miRNA reduction over a broader range of miRNA concentrations in the cell (Denzler et al., 2016).

### Argonaute Structural Considerations During TDMD

Argonaute proteins contain specific protein pockets that bind and protect the otherwise vulnerable 5′ and 3′ unmodified termini of miRNAs (Ma et al., 2005; Parker et al., 2005; Nakanishi et al., 2012, 2013; Faehnle et al., 2013; Schirle et al., 2014). This vulnerability precludes the accumulation of free mature miRNA in the cytoplasm. It thus seems reasonable to assume that the mechanism leading to miRNA-specific recognition and degradation must operate on AGO-loaded miRNAs. The fact that TDMD requires extensive complementarity between miRNAs and their target RNAs may be due to the AGO structural rearrangements that occur upon extensive, but not seed-matched target binding (**Figure 3B**). An extensive complementarity with a target RNA might detach the otherwise inaccessible 3′ end of the miRNA from its binding pocket at the AGO PAZ domain, therefore exposing it to the tailing and exonucleolytic enzymes (Lingel et al., 2003, 2004; Yan et al., 2003; Wang et al., 2008) (reviewed in Sheu-Gruttadauria and MacRae, 2017). Alternatively, as previously suggested (Ameres and Zamore, 2013), all miRNAs may be susceptible to tailing and trimming when bound to target RNAs, but the slower dissociation of only miRNAs with high-affinity sites might turn highly complementary RNAs more effective at triggering modification and degradation of bound miRNAs. Interestingly, current *in vitro* evidence might support the latter mechanism though in a miRNA-specific manner. Depending on the relative sequence composition of the seed vs. the 3′ region of the miRNA, such as its GC content, the dissociation rate of some miRNAs seems susceptible to the extent of pairing to their target RNA. For instance, AGO2 molecules loaded with let-7 miRNA dissociate at similar rates from targets bound by either seed-only or seed plus four additional 3′ supplementary base pairs (Wee et al., 2012). In contrast, dissociation of AGO2 loaded with miR-21, which features a more AU-rich seed than let-7, is slowed more than sevenfold upon 3′ supplementary base pairing relative to seed-only pairing (Salomon et al., 2015). Such differences in dissociation rates based on their internal sequence composition could render some miRNAs more susceptible to TDMD than others. Nevertheless, sequence-specific interactions are not the only determinants of the kinetics in target RNA binding by AGOs, as more complex sequence-independent contacts also affect binding kinetics and could equally influence TDMD efficacy (Ameres et al., 2007; Salomon et al., 2015).

### Shaping of miRNA Binding Sites and Specialization of Argonaute Proteins

Only unmodified miRNAs loaded on *Drosophila* AGO1 are affected by TDMD, while a minority of miRNAs that partition into *Drosophila* AGO2 bear a 2′-*O*-methyl group at their 3′ ends (Horwich et al., 2007; Pelisson et al., 2007) that renders them immune to TDMD. Similarly, endogenous siRNAs (endo-siRNAs), which are the main class of AGO2-bound small RNAs, show essentially full complementarity to cellular and transposon-derived mRNAs and are also both 2′-*O*-methylated and resistant to TDMD. As described for plants, only when de-protected in methylation deficient flies (*hen1* mutants), are siRNAs susceptible to TDMD. The fact that metazoan miRNAs lack 2′-*O*-methylation at their 3′ ends (Ameres et al., 2010; Ji and Chen, 2012) and that TDMD is restricted to unmodified and thus unprotected miRNAs, provides an evolutionary explanation by which TDMD might have shaped miRNA target sites toward a partial complementarity against animal miRNAs (Ameres et al., 2010; Ameres and Zamore, 2013). At the same time, by preventing TDMD on siRNAs, methylation by HEN1 might have contributed to the specialization of *Drosophila* AGO proteins, as a way to discriminate self from non-self siRNA targets (Forstemann et al., 2007; Ameres et al., 2010, 2011). In analogy to the functional specialization of AGO1 and AGO2 in flies, it is attractive to speculate that a functional specialization among AGO proteins in mammals—which are altogether closer in evolution to *Drosophila* miRNA-specific AGO1 than to siRNA-specific-AGO2 (Hutvagner and Simard, 2008; Swarts et al., 2014)—could have also occurred, maybe by conferring differential susceptibility to TDMD on the loaded miRNAs. Although current evidence might not support this possibility (Lee et al., 2013), it has not been exhaustively tested experimentally.

## Cell-Type Influence on TDMD Efficacy

Current evidence suggests that neurons trigger a more potent TDMD response in comparison to non-neuronal cells (de la Mata et al., 2015). For instance, artificial highly complementary targets have been shown to induce up to 10-fold reduction on miR-132 levels in neurons, while only about 2-fold in HEK293T cells. Similarly, while *Cyrano*-mediated TDMD decreases miR-7 levels by about 45-fold in neurons, *Cyrano* fragments containing either a wild-type miR-7 site or artificial sites extensively complementary to other miRNAs, induce only a modest reduction of about 3-fold on the cognate miRNAs in HEK293T cells (Kleaveland et al., 2018). Although the molecular basis of this phenomenon remains to be determined, it might reflect the generally higher miRNA metabolism observed in these cells (Rajasethupathy et al., 2009; Sethi and Lukiw, 2009; Krol et al., 2010a). At the same time, elevated TDMD activity in neurons appears well matched to the extensive dependence of the nervous system on highly dynamic and spatially localized regulation (Holt and Schuman, 2013). Nevertheless, other reports have shown that certain miRNAs are also short-lived in fibroblasts (Rissland et al., 2011; Marzi et al., 2016). Intriguingly, fast decaying miRNAs are associated with high stoichiometric target:miRNA ratios (Marzi et al., 2016) or even regulated by TDMD in this cell type (Ghini et al., 2018). Furthermore, all reported cases of viral TDMD achieve high efficiency in non-neuronal cells, indicating that the core of the TDMD machinery is not exclusive to neurons. Therefore, whether specific cell types maintain a constitutively more active TDMD machinery by relying on tissue-specific TDMD machinery remains to be determined.

## Functional Implications of TDMD

### Uncoupling miRNA Clusters

**FIGURE 4:**
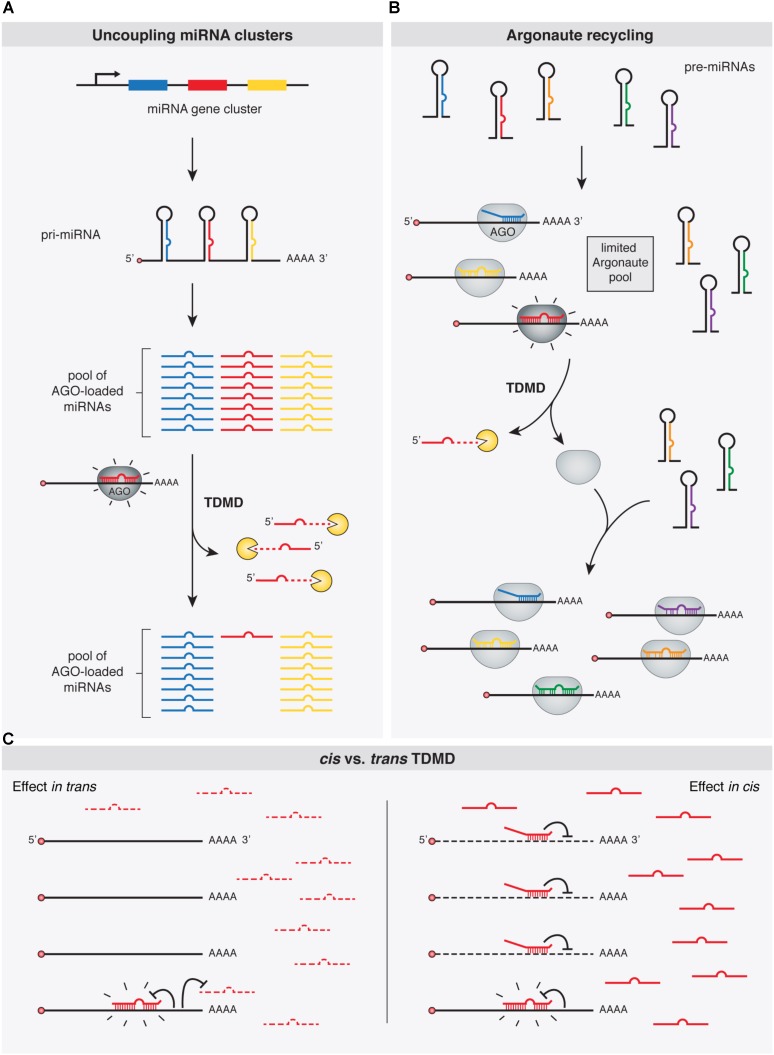
Functional implications of TDMD. TDMD might control and diversify miRNA expression patterns by means of: **(A)** allowing expression of specific miRNA family members from single gene clusters, e.g., in a tissue- or developmental time-specific manner; **(B)** promoting the recycling of Argonaute (Ago) proteins and thus the loading of newly synthesized miRNAs; or **(C)** Right panel: avoiding miRNA silencing *in cis*, e.g., under circumstances where TDMD-competent target RNAs might be too scarce to globally affect miRNA abundance *in trans*, but effective enough to trigger TDMD *in cis* and becoming immune to miRNA silencing (in contrast to the left panel where an abundant target RNA would additionally deplete a specific miRNA *in trans* globally in the cell, as in **A**). Shine emphasis is shown on “TDMD-primed” Ago-miRNA-target complexes.

A prevalent feature of miRNAs is that they are often closely clustered within the genome. In fact, over 30% of human miRNAs are predicted to be encoded within polycistronic miRNA clusters (Altuvia et al., 2005). In this context, an aspect of TDMD that seems to be particularly important is its ability to ‘uncouple’ miRNA clusters that are co-transcribed from a genomic locus as single primary transcripts (**Figure 4**). By depleting specific miRNAs, TDMD allows for differential expression of members within clusters in different conditions or cell types. For example, miR-27, together with miR-23 and miR-24, are derived from the same primary transcript, but only miR-27 is specifically targeted through TDMD during HVS or MCMV infections. Most notably, HCMV infection initially induces the transcription of all members in the *pri-Mir17-92* cluster, while TDMD targets only the miR-17 family. TDMD thereby enables HCMV to simultaneously up-regulate four members (miR-18a, miR-19a, miR-19b, and miR-92a) and down-regulate two members (miR-17 and miR-20a) of the same cluster. Yet, the adaptive value of establishing such specific expression domains for different miRNA family members is not immediately clear. This is because clusters often include several members of one or more miRNA families that share an identical extended seed region (miRNA nucleotides 2–8), the primary determinant of miRNAs targeting properties (Bartel, 2009). Therefore, all members within a family of miRNAs encoded in a given cluster, potentially regulate an overlapping set of targets (Bartel, 2009). Alternatively, differences in seed-distal miRNA pairing (**Figure 1**) may direct the different miRNA family members to distinct, only partially overlapping sets of targets (Moore et al., 2015; Broughton et al., 2016). At the moment, however, it remains to be determined whether the relevant biological outcome of TDMD—when involved in depleting unique miRNAs from co-expressed family members—is to reduce global expression of the whole family below certain thresholds or to modify the targeting specificity of the cluster. This is indeed the situation for mammalian *Nrep* mRNA in regulating miR-29b, one of the three members of the miR-29 family. While *Nrep* TDMD activity might lead to a sufficient reduction of the overall levels of the miR-29 family miRNAs rather than depletion of miR-29b specifically, it might also be that the seed-distal miRNA pairing of miR-29b may differ from the other miR-29 family members, reorienting silencing toward a different set of targets. The identification of new phenotypically relevant targets will ultimately provide a clearer picture.

### Argonaute Recycling

MiRNAs exist in the cell in association with AGO proteins, the core components of the RISC complex. Although there is evidence suggesting that miRNAs might be expressed in excess relative to AGOs in certain cell types (Janas et al., 2012; Stalder et al., 2013), the consensus view appears to be that miRNA steady state levels are the result of a stabilizing effect conferred by limiting amounts of AGOs binding to miRNAs, supporting the notion that most miRNAs detected in cells are loaded and stabilized by AGOs (Grishok et al., 2001; Diederichs and Haber, 2007; Khan et al., 2009; Krol et al., 2010b). In a context where miRNAs compete for limiting amounts of cellular AGO proteins, depletion of one miRNA by TDMD might additionally facilitate loading of other miRNAs (Hausser et al., 2013) (**Figure 4**). In this way, TDMD-mediated control of miRNA levels might be particularly useful to diversify and rapidly alter miRNA expression patterns. By accelerating the recycling of a miRNA in specific contexts, TDMD would help to avoid unsuitable activity of this miRNA. This appears particularly relevant because of miRNAs’ natural long half-lives, which would otherwise prevent their rapid downregulation through repression of their transcription (Hausser et al., 2013).

### Avoiding miRNA Silencing *in cis*

All the described potential functions of TDMD would implicate target RNAs acting *in trans*, thereby leading to a global reduction of given miRNAs in the cell. However, this might not always be the case. Instead, TDMD could conceivably operate *in cis* on certain targets by rendering them immune to silencing by particular miRNAs that could otherwise have detrimental effects on such targets. In fact, mutating the TDMD-competent miR-7 binding site present on the *Cyrano* lncRNA does not seem to influence *Cyrano* levels (Kleaveland et al., 2018), which is consistent with this idea. However, it should be noted that for *Cyrano* in particular another explanation could be invoked: a putatively abrogated repression at the mutant—formerly TDMD-competent—miR-7 site could be offset by enhanced repression (as a consequence of increased levels of miR-7 in the mutants) at a second, canonical site located at the beginning of *Cyrano* exon 3, which would refute the idea of TDMD playing a role *in cis* (Kleaveland et al., 2018). Nevertheless, it is tempting to speculate that a role *in cis* for TDMD could also operate, for example, on “over-conserved sites” which have been evolutionarily selected for some miRNA-independent reason even before the cognate miRNA appeared in evolution. Such exaptation events could have also occurred for canonical silencing by miRNAs. However, as previously suggested, because this kind of regulatory adaptation has yet never been demonstrated, it should be considered with caution (Pinzon et al., 2017; Seitz, 2017).

## Conclusion and Perspectives

The TDMD pathway has emerged as a means to specifically and dynamically regulate miRNA levels, adding another layer of regulation on small RNA stability that operates on top of RNA-binding proteins and *cis*-elements present on small RNA sequences (reviewed in Ji and Chen, 2012). Importantly, we are only just beginning to understand the physiological functions of TDMD which, we expect, will inspire further research into the prevalence and mechanisms of TDMD. Future challenges will include determining whether this is a relatively widespread mechanism or if it is only restricted to a few, limited cases. The fact that some viruses have co-opted TDMD for their own benefit, together with TDMD’s apparent conservation among several species, suggests that more instances of endogenous TDMD will be discovered, allowing us to discern the full adaptive value of TDMD.

## Permission to reuse and Copyright

Permission must be obtained for use of copyrighted material from other sources (including the web). Please note that it is compulsory to follow figure instructions.

## Author Contributions

All authors contributed with discussing and writing different sections of the manuscript. MdlM coordinated and assembled the final version of the manuscript.

## Conflict of Interest Statement

The authors declare that the research was conducted in the absence of any commercial or financial relationships that could be construed as a potential conflict of interest.
